# Tranexamic acid as an alternative to epinephrine in WALANT carpal tunnel release: a randomized clinical trial

**DOI:** 10.1186/s12893-026-03976-z

**Published:** 2026-07-03

**Authors:** Lincoln Kamimura, Martha Cecilia Castano-Betancourt, Marcelo Claudiano de Castro, Cristiano Almeida Bastos, Carolina Isper, Ewerton Alexandre Galdeano, Helton Hiroshi Hirata

**Affiliations:** 1 Postgraduation Department, Faculdade de Medicina de Jundiaí, Jundiai, Sao Paulo, Brazil; 2 Orthopedic Department, Hospital de Caridade São Vicente de Paulo (HCSVP), Jundiaí, Brazil

**Keywords:** Hand, Carpal tunnel syndrome, Local anesthesia, Surgical complications, Postoperative, Clinical trial

## Abstract

**Background:**

Carpal tunnel release can be performed using the WALANT (Wide-Awake Local Anesthesia No Tourniquet) technique, in which epinephrine is commonly used to improve intraoperative hemostasis. However, concerns regarding vasoconstrictor-related complications persist. This study aimed to compare tranexamic acid (TA) with epinephrine for bleeding control during WALANT carpal tunnel release.

**Methods:**

A prospective randomized study including 53 patients with carpal tunnel syndrome was conducted. Patients were randomized to WALANT with epinephrine (*n *= 27) or tranexamic acid (*n *= 26). Surgical time, intraoperative hemostasis, patient satisfaction, and postoperative complications were recorded. Clinical outcomes were assessed using specific clinical tests and the Boston Carpal Tunnel Questionnaire preoperatively and at 30 days.

**Results:**

No severe bleeding or ischemic complications were observed in either group. One infection occurred in the epinephrine group requiring surgical debridement. Greater difficulty in achieving intraoperative hemostasis was observed in the TA group, resulting in longer surgical times (29.3 ± 10.5 min vs. 21.4 ± 6.9 min; *P *< 0.001). Patient satisfaction and functional outcomes were high and comparable between groups.

**Conclusion:**

Carpal tunnel release performed using the WALANT technique with either epinephrine or tranexamic acid was associated with similar short-term functional improvement and patient satisfaction. No major complications were observed in either group. However, the use of tranexamic acid was associated with greater difficulty in achieving intraoperative hemostasis and longer surgical times.

**Trial registration:**

The trial protocol is available in the trial registry at Brazilian Clinical Trials Registry (ReBec) under registration number RBR2njtg49, date of registration 11/07/2025 (retrospectively registered). https://ensaiosclinicos.gov.br/rg/RBR2njtg49.

## Background

Carpal tunnel syndrome (CTS) is the most common compressive neuropathy of the upper limb and a frequent cause of pain, numbness, and functional limitation of the hand [1]. It results from compression of the median nerve within the carpal tunnel at the wrist and can significantly affect quality of life and work capacity. When conservative treatment fails, carpal tunnel release (CTR) surgery is considered the most effective treatment and is widely performed worldwide [2].

Different anesthesia techniques have been used for CTR, including regional anesthesia, monitored anesthesia care, and local anesthesia with a tourniquet. In recent years, the WALANT (Wide-Awake Local Anesthesia No Tourniquet) technique has gained increasing popularity in hand surgery [3]. This approach allows procedures to be performed using local anesthesia without sedation or a tourniquet, improving patient comfort and reducing the need for operating room resources or other complications such as tourniquet-related pain, transient paresthesia, and risks associated with general anesthesia or regional nerve blocks [4–7].

In the traditional WALANT technique, epinephrine is added to the anesthetic solution to provide vasoconstriction and improve intraoperative hemostasis [4]. Although this method has been shown to be safe and effective, concerns remain regarding the use of vasoconstrictors in the hand because of the potential risk of digital ischemia, even though this complication is rare.

Tranexamic acid (TA) is an antifibrinolytic agent widely used to reduce bleeding in several surgical specialties and is included in the list of essential medicines of the World Health Organization [8]. Because TA stabilizes blood clots without causing vasoconstriction, it may represent an alternative strategy for bleeding control during WALANT procedures.

Although substantial evidence supports the use of TA in major orthopedic surgeries, its application in minor hand procedures, such as carpal tunnel release, remains less well established. Evidence regarding its effectiveness for hemostasis and its potential complications in these procedures is still limited.

Therefore, the aim of this randomized clinical trial was to compare the effectiveness of epinephrine and tranexamic acid for bleeding control during carpal tunnel release performed using the WALANT technique. In addition to evaluating bleeding control, the study assessed safety outcomes, including bleeding-related complications, postoperative infection requiring surgical intervention, ischemic events, and the need for intraoperative conversion to the traditional technique with tourniquet use. Secondary outcomes included comparison of surgical time between groups and assessment of intraoperative difficulty in achieving adequate hemostatic control.

## Methods

This prospective randomized study was conducted at a reference center for orthopedics and traumatology in a high-complexity hospital. The protocol for this study was approved by the appropriate institutional review committee and meet the guidelines of their responsible governmental agency. Our manuscript adheres to CONSORT guidelines (http://www.consort-statement.org/) for reporting clinical trials.

Eligible participants were adults (≥ 18 years) with American Society of Anesthesiologists (ASA) physical status I or II scheduled for elective carpal tunnel release surgery.

Exclusion criteria included:

- age < 18 years.
- contraindications to tranexamic acid (TA), including renal insufficiency, history of seizures, or vascular insufficiency.
- cognitive impairment or anxiety disorders that could interfere with the surgical technique.
- inability to provide informed consent.

### Randomization and blinding

Participants were randomized in a 1:1 ratio using blocked randomization to ensure balanced group sizes (*N* = 53). The random allocation sequence was generated using the random number function in Microsoft Excel. Blocked randomization was used to maintain balance between groups, and the block size was not disclosed to the surgeons involved in the procedures.

The allocation sequence was prepared by a researcher who was not involved in patient recruitment, intervention delivery, or outcome assessment. Group assignments were placed in sequentially numbered, opaque, sealed envelopes. After patient enrollment, the next envelope in sequence was opened to determine group allocation, ensuring concealment of the allocation sequence until assignment.

Surgeons were blinded to the block size and allocation sequence. Outcome assessors were blinded to the intervention group; however, participants were aware of their assigned technique due to the nature of the intervention.

### Interventions

Both interventions were performed under the same operative conditions and perioperative protocols to ensure comparability between groups, with the surgical technique representing the only difference between interventions. Participants were allocated into two groups:

### Group 1 – WALANT with epinephrine

The traditional WALANT technique was performed using 0.5% lidocaine combined with epinephrine (1:100,000) and buffered with sodium bicarbonate.

### Group 2 – WALANT with tranexamic acid

A modified WALANT technique was used with 0.5% lidocaine without vasoconstrictor. Participants received 1 g of intravenous tranexamic acid before surgical incision. After tissue dissection, a topical solution containing 1 g of TA diluted in 100 mL of 0.9% saline was applied to the surgical field for 5 min, followed by removal with sterile gauze.

At the end of the study, 27 patients were allocated to Group 1 and 26 to Group 2. Analyses were performed according to the intention-to-treat principle.

### Preoperative counseling

Participants in Group 1 were informed about the possibility of transient digital ischemia associated with epinephrine use. Participants in Group 2 were counseled regarding the potential limitations of TA in controlling intraoperative bleeding and the possible need for sedation, tourniquet use, or general anesthesia if bleeding control was inadequate.

### Surgical procedure

All procedures were performed by experienced orthopedic surgeons specialized in hand surgery.

Patients were positioned supine, and antisepsis of the hand, forearm, and upper arm was performed using 2% chlorhexidine followed by alcoholic chlorhexidine solution. A non-inflated tourniquet was positioned as a safety precaution.

Procedures were performed without sedation, although equipment for sedation and general anesthesia was available if required.

Local anesthesia was administered by the surgeon using a fine needle (13 × 4.5 mm). A total of 20 mL of anesthetic solution was infiltrated subcutaneously:

- 10 mL between the ulnar and median nerves (approximately 5 mm proximal to the wrist crease and 5 mm ulnar to the median nerve)
- 10 mL at the incision site

The needle was inserted perpendicular to the skin while maintaining at least 5 mm distance from the nerve structures.

Operating room staff were instructed to maintain neutral communication during the procedure to minimize patient anxiety.

### Postoperative care

Following surgery, the operated limb was immobilized and patients were monitored for at least three hours to assess digital perfusion, bleeding, and hematoma formation.

In Group 1, phentolamine was available for reversal of epinephrine-induced ischemia if necessary.

Follow-up visits were scheduled at 7 days and 30 days postoperatively.

### Outcomes and data collection

#### Demographic and clinical variables

The following baseline demographic and clinical variables were collected and analyzed: age, sex, affected side, duration of symptoms, comorbidities, and the presence of isolated carpal tunnel syndrome (CTS) or CTS associated with Lacertus syndrome. These variables were recorded to characterize the study population and to explore potential differences between the intervention groups.

#### Surgical outcomes

The following surgical outcomes were recorded: operative time, quality of the surgical field based on intraoperative bleeding control and visualization of anatomical structures, the need for conversion or interruption of the planned technique due to difficulty controlling bleeding, and patient satisfaction with the procedure. These measures were used to compare the effectiveness of hemostasis and the overall surgical performance between the two study groups.

#### Complications

Postoperative complications were monitored and recorded during follow-up. These included severe bleeding, digital ischemia, infection, suture dehiscence, tissue necrosis, paresthesia, and stiffness affecting the hand or finger joints. Any adverse events occurring during the postoperative period were documented to evaluate the safety of the two hemostatic strategies.

#### Functional outcomes

Functional outcomes were assessed using the Boston Carpal Tunnel Questionnaire (BCTQ) before surgery and 30 days postoperatively [8]. The Boston Carpal Tunnel Questionnaire (BCTQ) was used to assess symptom severity and functional status. The instrument consists of two subscales: the Symptom Severity Scale (SSS) and the Functional Status Scale (FSS), with higher scores indicating greater symptom severity and functional impairment.

The BCTQ evaluates:

- Symptom Severity Scale (SSS) – 11 questions
- Functional Status Scale (FSS) – 8 questions

Each item is scored from 1 (no symptoms/difficulty) to 5 (severe symptoms/difficulty). Mean scores for each scale were calculated [9].

The minimal clinically important difference (MCID) for the BCTQ was considered when interpreting postoperative changes. A minimum difference of 0.8 in the mean change of the Symptom Severity Scale and 0.5 in the Functional Status Scale (FSS) between pre- and postoperative scores has been reported as clinically meaningful [10].

#### Clinical tests for CTS

Three diagnostic tests were performed:

- Tinel’s sign: percussion over the median nerve producing paresthesia [11].
- Phalen’s test: wrist flexion for 60 s producing symptoms [12].
- Durkan’s test: direct compression of the median nerve for 30 s [13].

#### Sample size

The sample size corresponded to all eligible patients treated during the study period according to the study protocol.

### Statistical analysis

The study aimed to compare postoperative complications and functional outcomes between patients undergoing WALANT carpal tunnel release using epinephrine or tranexamic acid. Continuous variables were analyzed using two-tailed Student’s t-tests, while categorical variables were compared using Chi-square or Fisher’s exact tests. Analysis of variance (ANOVA) was performed to evaluate differences between groups. A p-value < 0.05 was considered statistically significant. Results are reported with 95% confidence intervals (CI). All analyses were performed using SPSS version 25. Data were analyzed according to the intention-to-treat principle. All randomized participants completed the 30-day postoperative follow-up and had complete outcome data; therefore, no missing data occurred and no imputation methods were required.

#### Interim analyses

No interim analyses or stopping guidelines were planned.

#### Protocol changes

No important changes to the trial methods, outcomes, or analyses were made after trial commencement.

#### Patient and public involvement

Patients and the public were not involved in the design, conduct, reporting, or dissemination plans of this research. Participants were involved only as subjects in the study and attended scheduled follow-up assessments.

## Results

**Fig. 1:**
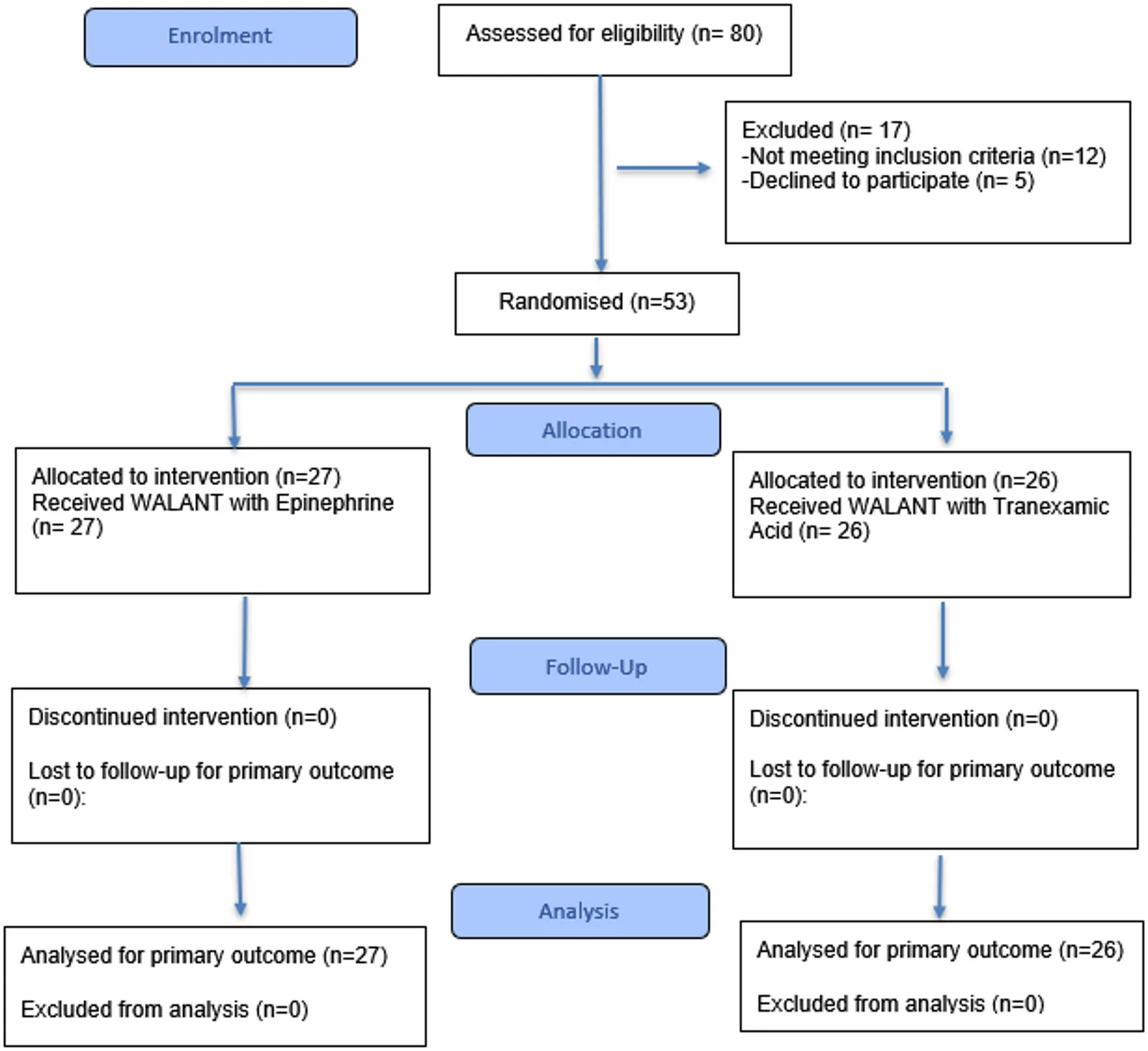
Flow diagram (CONSORT 2025) of the progress through the phases of this randomised trial of two groups

Figure 1 shows the CONSORT 2025 flowchart. A total of 53 patients with carpal tunnel syndrome (CTS) who underwent surgical treatment between December 2022, and April 2023 were included.

### Baseline characteristics

Baseline characteristics of the study population are presented in Table 1. The mean age was 52.1 ± 8.9 years in Group 1 (vasoconstrictor) and 56.8 ± 11.3 years in Group 2 (tranexamic acid, TA), with no statistically significant difference between groups (*P* = 0.10). Most participants were women (89%), and 34% reported experiencing carpal tunnel syndrome (CTS) symptoms for four years or longer.

**Table 1:** Demographic data of patients who participated in this study and preoperative variables

Characteristics	Group 1: WALANT - Epinephrine (*n* = 27)	Group 2: WALANT - Tranexamic Acid (*n* = 26)
Age	52.1 ± 8.94	56.8 ± 11.3
Sex		
Male	1 (3.7)	5 (19.2)
Female	26 (96.3)	21 (80.8)
Diagnosis		
Carpal tunnel syndrome (CTS)	26 (96.3)	24 (92.3)
CTS + Lacertus	1 (3.7)	1 (3.8)
Affected side		
Right	18 (66.7)	17 (65.4)
Left	9 (33.3)	9 (34.6)
Duration of symptoms		
2 years or less	10 (37)	10 (38.5)
3 years	7 (25.9)	7 (26.9)
4 years	10 (37)	8 (30.8)
Number of comorbidities	1.11 (1.08)	1.23 (1.08)
Hypertension	12 (44.4)	13 (50)
Diabetes Mellitus	10 (37)	5 (19.2)
Hypothyroidism	3 (11.1)	4 (15.4)
Preoperative tests:		
Tinel (positive)	23 (85.2)	26 (100)
Durkan (positive)	23 (85.2%)	23 (88.5%)
Phalen (positive)	22 (81.5)	24 (92.3)
Lacertus (positive)	3 (11.1)	4 (15.4)
Median itch test (positive)	0 (0)	1 (3.8)
Preoperative Symptoms Severity Scale (Boston Q.)ɫ	3.75 (0.13)	3.57 (0.13)
Preoperative Functional Status Scale (Boston Q.)ɫ	3.33 (0.17)	2.98 (0.17)

Univariate Analysis (ANOVA). Values are number (N) with percentages (%) or mean ± Standard Error (SE) = by the acronym in English Standard Error. ɫ. *Abbreviations*: *TA* Tranexamic Acid, *Boston Q* Boston Questionnaire

There were no significant differences in baseline demographic or clinical characteristics between the two groups. Preoperative CTS symptoms, functional scores measured by the Boston Carpal Tunnel Questionnaire (BCTQ), diagnostic CTS tests, and the prevalence of comorbidities were also comparable between groups (Table 1).

### Primary outcome: complications

The primary outcome was the occurrence of complications, particularly severe or life-threatening bleeding. No patient experienced severe bleeding during the study period.

One participant in the vasoconstrictor group developed a postoperative infection that required surgical debridement. No ischemic events were observed, and there were no cases requiring conversion to the conventional technique with tourniquet use.

### Surgical time

Mean surgical time was 29.3 min in the TA group and 21.4 min in the vasoconstrictor group (Table 2). Intraoperative observations suggested greater difficulty achieving adequate hemostasis in the TA group, requiring more frequent pauses for bleeding control and resulting in reduced visualization of the surgical field.

### Postoperative diagnostic tests

Postoperative CTS diagnostic tests showed similar results between groups. The Phalen test was borderline significant in favor of the vasoconstrictor group, in which all patients tested negative (*P* = 0.07), although the number of positive tests in the TA group was also low (Table 2).

The proportion of patients demonstrating improvement in CTS diagnostic tests between baseline and the 30-day follow-up was comparable between groups. Postoperative Symptoms and Functional Outcomes (BCTQ).

At 30 days postoperatively, there were no significant differences between the two groups in BCTQ symptom severity scores or functional status scores (Table 2, *P* > 0.05).

Postoperative BCTQ scores were similar between groups for both symptom severity (*P* = 0.97) and functional status (*P* = 0.97). The change in symptom severity scores from baseline to postoperative assessment was also comparable between groups (*P* = 0.47), while the difference in functional status scores approached borderline significance (*P* = 0.09).

Overall, both groups demonstrated improvement in BCTQ scores compared with baseline, indicating reduction of symptoms and improvement in hand function following surgery.

### Patient satisfaction

Patient satisfaction after surgery was high and similar between groups (Table 2). The mean satisfaction score was 7.96 (95% CI 7.2–8.7) in the TA group and 8.35 (95% CI 7.6–9.1) in the vasoconstrictor group (*P* = 0.47).

**Table 2:** Effect of tranexamic acid on results after 30 days

Postoperative variables and results	Group 1: WALANT - Epinephrine (*n* = 27)	Group 2: WALANT - Tranexamic Acid (*n* = 26)	*P*
Postoperative Complication Infection No. (%)‡	1 (3.7)	0 (0)	N.A
Operative Time (minutes) Mean (95%CI)	21.4 (20.3–22.6)	29.3 (28.1–30.5)	< 0.001
Postoperative Exams:			
Tinel (positive)	1	3	0.3
Durkan (positive)	0	2	0.14
Phalen (positive)	0	3	0.07
Lacertus (positive)	0	1	0.30
Itch Test No. Median	0	0	NA
N. Patients with improvement in CTS tests (%)			
Tinel Positive Difference	22 (81.5)	23 (88.5)	0.78
Durkan Positive Difference	23 (85.2)	21 (80.8)	0.67
Phalen Positive Difference	22 (81.5)	21 (80.8)	0.95
Lacertus Positive Difference	3 (11.1)	3 (11.5)	0.96
Itch Test Positive Difference	0 (0)	1 (3.8)	0.30
BCTQ scores after surgery Mean ± 95% CI			
POP Symptom Severity Scale (Boston Q.)ɫ	1.65 (1.39–1.91)	1.64 (1.38–1.91)	0.97
POP Functional Status Scale (Boston Q.)ɫ	1.83 (1.55–2.12)	1.85 (1.56–2.14)	0.95
Difference between pre- and postoperative symptom scores	2.1 (1.77–2.44)	1.93 (1.58–2.27)	0.47
Difference between pre- and postoperative functional score	1.50 (1.20–1.79)	1.13 (0.83–1.43)	0.09
Postoperative satisfaction (0 = Dissatisfied/10 = Very satisfied)	8.35 (7.6–9.1)	7.96 (7.2–8.7)	0.47
Secondary outcomes - n. (%)			
MCID Symptoms	25 (92.6)	22 (84.6)	0.36
MCID Function	23 (85.2)	22 (84.6)	0.95

Positive difference: number of patients showing improvement in carpal tunnel syndrome (CTS) diagnostic tests between baseline and the 30-day postoperative evaluation

MCID: minimal clinically important difference for the Boston Carpal Tunnel Questionnaire (BCTQ); values ≥ 0.8 for the Symptom Severity Scale and ≥ 0.5 for the Functional Status Scale are considered clinically meaningful

## Discussion

In this randomized study, tranexamic acid and epinephrine were associated with similar short-term functional outcomes and patient satisfaction, although greater difficulty in achieving intraoperative hemostasis and longer operative times were observed in the tranexamic acid group. While epinephrine is routinely used to provide intraoperative hemostasis during WALANT procedures, concerns persist regarding vasoconstrictor-related complications and its use in selected patient populations [14–16]. Therefore, identifying effective alternatives for bleeding control remains clinically relevant.

In the traditional WALANT technique, epinephrine is added to the anesthetic solution to induce vasoconstriction and improve intraoperative bleeding control [16–19]. Although this approach eliminates the need for a tourniquet, it requires additional preparation, including buffering the solution with sodium bicarbonate and allowing time, typically around 30 min for the vasoconstrictive effect to take place. These factors may impact surgical workflow and efficiency in certain clinical settings.

In the present randomized study, both epinephrine and tranexamic acid (TA) used with local anesthesia were safe for carpal tunnel release surgery, with no severe bleeding or ischemic complications observed. However, greater difficulty in intraoperative hemostatic control was noted in the TA group, resulting in longer surgical times compared with the vasoconstrictor group. The longer operative times observed in the TA group may reflect differences in the mechanism of action of the two agents. Whereas epinephrine provides local vasoconstriction, tranexamic acid acts by inhibiting fibrinolysis without affecting vascular tone [16–19]. This approach eliminates the need for a tourniquet but requires an additional preparation step, since the pH of the anesthetic solution must be balanced with sodium bicarbonate. In addition, surgeons usually wait about 30 min before starting the procedure to allow the vasoconstrictive effect to occur.

The greater difficulty in achieving hemostasis observed in the tranexamic acid group may be explained by differences in the mechanisms of action of the two agents. Whereas epinephrine provides local vasoconstriction, tranexamic acid acts by inhibiting fibrinolysis without affecting vascular tone [20–22]. Consequently, diffuse bleeding at the surgical site may be more difficult to control, potentially contributing to the longer operative times observed in the tranexamic acid group [23, 24].

Despite these differences in bleeding control, no cases of severe or life-threatening bleeding were observed in either group. Sasor et al. reported minimal differences in blood loss during carpal tunnel release performed with WALANT compared with procedures using a tourniquet, with only about 1 mL more blood loss in the WALANT group [23]. In the present study, bleeding volume was not quantified, which represents a limitation of our analysis.

Only one patient in the vasoconstrictor group developed a postoperative infection requiring surgical debridement. Although WALANT procedures are sometimes performed with less extensive sterility setups, current evidence has not demonstrated higher infection rates when this technique is used.

“Concerns regarding the safety of local anesthesia remain a barrier to the adoption of WALANT for carpal tunnel release, although serious complications appear to be uncommon. Local anesthetic systemic toxicity (LAST) is a potentially severe adverse event; however, no cases associated with WALANT carpal tunnel surgery have been reported in the literature [25, 26]. Epinephrine may also cause transient adverse effects such as palpitations, vasovagal reactions, or arrhythmias [4]. None of these events were observed in the present study. Nevertheless, the relatively small sample size limits the ability to detect uncommon or rare complications. Therefore, the absence of major adverse events should be interpreted cautiously and should not be interpreted as evidence that the safety profiles of the two interventions are equivalent.

Another concern related to epinephrine use is digital ischemia. Although there are a few case reports describing this complication after hand surgery, it remains extremely uncommon [4, 27]. In our study, no ischemic events were observed in either group.

Both groups in our study demonstrated significant improvement in pain and functional status 30 days after surgery compared with baseline Boston Carpal Tunnel Questionnaire scores. However, no significant differences were observed between the vasoconstrictor and TA groups. Patient satisfaction after surgery was high in both groups.

Previous studies have shown that WALANT is associated with pain levels that are similar to or lower than those observed with other anesthesia techniques, including procedures using tourniquets, monitored anesthesia care, or nerve blocks [3, 28, 29]. Similarly, Karamanis et al. reported that functional outcomes after carpal tunnel release performed under WALANT were not influenced by the type of local anesthetic used [28]. Our findings are consistent with these results.

Carpal tunnel syndrome is a common condition, and carpal tunnel release remains the only treatment with well-established long-term benefits. As patient-centered outcomes gain greater importance in healthcare evaluation, alternative anesthesia strategies that reduce costs while maintaining safety and patient satisfaction become increasingly relevant. Recent studies have also shown that WALANT procedures can be safely performed outside the traditional operating room, using field sterility in procedure rooms, which may improve efficiency and reduce healthcare costs [30–32]. Recent regulatory safety communications regarding tranexamic acid have primarily concerned inadvertent neuraxial administration resulting from medication errors rather than local infiltration techniques such as those used in the present study. Nevertheless, these reports underscore the importance of appropriate drug labeling, storage, and administration procedures when TXA is used in perioperative settings (FDA Provides Update to Health Care Professionals About Risk of Inadvertent Intrathecal (Spinal) Administration of Tranexamic Acid Injection | FDA 10/21/2025) [33].

This study has several limitations. First, the sample size was relatively small and may have been insufficient to detect uncommon adverse events. Second, intraoperative hemostatic control was assessed subjectively by the operating surgeon, and blood loss was not quantitatively measured. Consequently, differences in hemostatic efficacy between groups should be interpreted as differences in perceived surgical field control rather than quantified blood loss. Although operative time was significantly longer in the tranexamic acid group, suggesting greater difficulty in achieving adequate hemostasis, the magnitude of any difference in blood loss remains uncertain. Future studies should incorporate objective measures of intraoperative bleeding. Third, although the study was conducted prospectively, trial registration occurred after study initiation. This may increase the risk of reporting bias and limits independent verification that study outcomes and analyses were prospectively prespecified.

Finally, although patients were randomized, they were aware of the treatment they received because the potential risks and benefits of each method were explained before surgery and the outcomes were evaluated only 30 days after surgery, whereas many studies assessing carpal tunnel release report follow-up periods of six months or longer.

Another limitation is that patient-specific factors such as comorbidities or symptom duration were not specifically analyzed. These variables may influence bleeding control, healing, and postoperative recovery. Future studies including these factors may provide a more detailed understanding of the role of tranexamic acid in WALANT procedures.

## Conclusions

Carpal tunnel release performed using the WALANT technique with either epinephrine or tranexamic acid was safe and resulted in comparable short-term functional improvement and patient satisfaction. However, the use of tranexamic acid was associated with greater difficulty in achieving intraoperative hemostasis and longer surgical times.

While epinephrine remains more effective for rapid and efficient bleeding control, tranexamic acid may be considered a potential alternative in selected cases where the use of vasoconstrictors is contraindicated or undesirable. These findings support the feasibility of a vasoconstrictor-free WALANT approach, although with potential trade-offs in operative efficiency. Given the limited sample size, the study was not powered to detect rare adverse events or formally establish non-inferiority. Further studies with larger samples and longer follow-up are warranted to better define the comparative effectiveness and safety of these approaches in hand surgery.

## Data Availability

De-identified individual participant data, the data dictionary, and statistical code may be made available upon reasonable request to the corresponding author, subject to institutional approval and data sharing agreements.
